# Book Reviews

**Published:** 1898-09

**Authors:** 


### *BOOK REVIEWS, PAMPHLETS, EXCHANGES.*

---

### *BOOK REVIEWS.*

[Contributions solicited for review.]

---

We are in receipt of a complimentary copy of a small brochure entitled "A Country Doctor," by Dr. Thomas Hall Shastid, of Battle Creek, Mich. This is a plain but beautiful tribute to the life of a "country doctor" who has been the author's best friend, that is his father. The illustrations are very true to life and enhance very much the appearance of the book. The Doctor is to be congratulated upon the charming manner in which he presents the life of "A Country Doctor."

---

A Manual of Modern Surgery, General and Operative. By John Chalmers DaCosta, M.D., Clinical Professor of Surgery, Jefferson Medical College, Philadelphia. Published by W. B. Saunders, Philadelphia.

When we contemplate the great number of excellent works, both large and small, on the subject of Surgery, and see that in four years the author of the above work has had to issue a second edition, then that of itself speaks for the excellency of the book. Dr. DaCosta is comparatively a young man, and yet by his ability and energy has gained a foremost position as a surgeon. In this second edition a good many new subjects have been treated, which will still further enhance the value of the work. We believe that its continued success is assured. It is certainly a safe guide.

---

Atlas and Epitome of Operative Surgery. By Dr. Otto Zuckerkondl, of Vienna. With twenty-four colored plates and 217 illustrations in the text. Published by W. B. Saunders, Philadelphia. Price, $3.00 net.

This is another one of the popular atlases being published by the well-known house of W. B. Saunders. They are translations from the original German edition. In this case the American

Editor is Dr. Chalmers DaCosta, a man eminently fitted for such work. This character of a book is quite an innovation, but we must say that it commends itself to us. This especial Atlas on Operative Surgery is not quite so well gotten up in colored plates as some of the others, but the other illustrations are very good indeed. Such a work as this is extremely helpful to the student and even to the busy surgeon. To see an operation well illustrated, especially if in colors, is better understood by the reader than two pages of text. Indeed, such is often understood where reading the text produces confusion. As a quick reference book they are extremely valuable.

The subject of amputations is especially well treated, as is also the ligation of vessels. In fact, every portion of operative surgery is well represented. We predict a large sale for these works.

---

Lectures on Tumors. By John B. Hamilton, M.D., LL.D., Professor of Surgery, Rush Medical College and Chicago Policlinic; Consulting Surgeon to St. Joseph's Hospital, etc., etc., etc. Third Edition. Twenty-one Illustrations. Philadelphia. P. Blakiston's Son & Co. 1012 Walnut street. 1898. Price, $1.25 net.

This is a collection of the lectures of Dr. Hamilton to the students of the Medical Department of Georgetown University, which lectures, keeping pace with surgical knowledge, were repeated to the students of Rush Medical College. The book is in lecture form as heretofore, and its purpose is to aid students in their preparations for recitation, though of course this does not impair its value as a reference for practitioners.

The introduction is a brief description of some methods of preparing sections for microscopic examination—a very useful part of the book. The appendix gives the Rush Medical College methods.

"General considerations" deal with the features which must be borne in mind in diagnosis. The classification is that of the Royal College of Physicians, 1896, though it is not claimed to be perfect. The different tumors are next treated in detail. His description of "Condyloma" is rather obscure. He is rather too willing to accept the (alleged) parasite of epithelioma. The brevity and practicality of the little work should recommend it to the busy practitioner. M. B. H.

THE OFFICE TREATMENT OF HEMORRHOIDS, FISTULA, ETC., WITHOUT OPERATION. Together with Remarks on the Relation of Diseases of the Rectum to Other Diseases in Both Sexes, Especially in Women, and the Abuse of the Operation of Colostomy. By Charles B. Kelsey, A.M., M.D., New York.

The subjects treated comprise three lectures delivered by the author to his class, and have reached us from the publisher, E. R. Pelton, No. 19 East Sixteenth street, New York.

The author gives a practical and common-sense view of the subject-matter of his little book, which makes it a valuable acquisition to the library, not only of the surgeon, but of every intelligent practicing physician. He shows that the usual and generally adopted surgical operation for the radical cure of piles is not always necessary, but it can be done by competent and skillful physicians without resort to the knife or cautery.

In the second lecture he points out the complications likely to exist in cases of piles in women, such as enlargement of the womb and prolapse of the ovary, and the futility of expecting a radical cure of piles without first relieving the uterine and ovarian troubles.

In the third lecture he condones colostomy (or making an artificial anus) as being the best means for the radical cure of stricture of the rectum from ulceration, thickening and contraction of the tissues, preferring removal of the diseased parts and making an artificial anus at the natural outlet. He emphasizes the danger of the operation in unskillful hands, but says that with the skill born of long practice it may be done as safely as colostomy.

We would advise all interested in the subjects treated to read this little book. G. G. R.

---

ATLAS OF SYPHILIS AND THE VENEREAL DISEASES, Including a Brief Treatise on the Pathology and Treatment. By Prof. Dr. Franz Mracek, of Vienna. Authorized Translation from the German. Edited by L. Bolton Bangs, M.D., Consulting Surgeon to St. Luke's Hospital and the City Hospital, New York, etc., etc. With 71 colored plates. Philadelphia. W. B. Saunders. Price $3.50, net. 1898.

The first three-fourths of this book is composed of illustrations of the various lesions of the genitals, skin, mouth, throat and other parts, reproduced mostly in colors. The picture occupies the right-hand page, while on the left, opposite, is given the history of the case, with satisfactory brevity and clearness. Many unusual lesions are found pictured and described, even those of great rarity. The colors are rather too vivid, but the illustrations as a whole are as nearly perfect as could be.

The remainder of the book is occupied with a condensed but very full description of syphilis and the venereal diseases. The author retains the old division of syphilis into primary, secondary, and tertiary. Treatment, both internal and external, is very thoroughly gone into. Simple ulcers, gonorrhea and its complications, and condyloma acuminata are next described, with treatment. M. B. H.

